# A Recognition Method of Aggressive Driving Behavior Based on Ensemble Learning

**DOI:** 10.3390/s22020644

**Published:** 2022-01-14

**Authors:** Hanqing Wang, Xiaoyuan Wang, Junyan Han, Hui Xiang, Hao Li, Yang Zhang, Shangqing Li

**Affiliations:** 1 College of Electromechanical Engineering, Qingdao University of Science & Technology, Qingdao 266000, China; 2019030020@mails.qust.edu.cn (H.W.); b021030001@mails.qust.edu.cn (J.H.); 2019030024@mails.qust.edu.cn (H.X.); 4019030012@mails.qust.edu.cn (H.L.); 4019030049@mails.qust.edu.cn (Y.Z.); 4019030017@mails.qust.edu.cn (S.L.); 2 Collaborative Innovation Center for Intelligent Green Manufacturing Technology and Equipment of Shandong Province, Qingdao 266000, China

**Keywords:** aggressive driving behavior, class imbalance dataset, ensemble learning, deep learning, advanced driver assistance system

## Abstract

Aggressive driving behavior (ADB) is one of the main causes of traffic accidents. The accurate recognition of ADB is the premise to timely and effectively conduct warning or intervention to the driver. There are some disadvantages, such as high miss rate and low accuracy, in the previous data-driven recognition methods of ADB, which are caused by the problems such as the improper processing of the dataset with imbalanced class distribution and one single classifier utilized. Aiming to deal with these disadvantages, an ensemble learning-based recognition method of ADB is proposed in this paper. First, the majority class in the dataset is grouped employing the self-organizing map (SOM) and then are combined with the minority class to construct multiple class balance datasets. Second, three deep learning methods, including convolutional neural networks (CNN), long short-term memory (LSTM), and gated recurrent unit (GRU), are employed to build the base classifiers for the class balance datasets. Finally, the ensemble classifiers are combined by the base classifiers according to 10 different rules, and then trained and verified using a multi-source naturalistic driving dataset acquired by the integrated experiment vehicle. The results suggest that in terms of the recognition of ADB, the ensemble learning method proposed in this research achieves better performance in accuracy, recall, and F_1_-score than the aforementioned typical deep learning methods. Among the ensemble classifiers, the one based on the LSTM and the Product Rule has the optimal performance, and the other one based on the LSTM and the Sum Rule has the suboptimal performance.

## 1. Introduction

Traffic accidents have been around since Karl Benz invented the car. With the development of society and economy, the number of cars is increasing, which has led to the increase in traffic congestion and traffic accidents. The research suggests that more than 90% of traffic accidents are caused by human factors [1]. Among them, a survey of the AAA Foundation for Traffic Safety shows that about 55.7% of fatal traffic accidents were associated with aggressive driving behavior (ADB) [2], and there is a positive correlation between ADB and the probability of traffic accidents [3,4]. As one of the main causes of traffic accidents, ADB is affected by situational factors such as traffic congestion [5,6] and personal factors such as negative emotions [7]. Due to the increasingly crowded traffic system and the accelerated pace of life, it is easier for drivers to exhibit ADB, so it is urgent to accurately recognize ADB. However, there is no uniform definition of ADB. ADB was mostly defined from the perspective of traffic psychology in existing studies as a syndrome of frustration-driven instrumental behaviors, that is, deliberately dangerous driving to save time at the expense of others [8]; the driving behavior that is likely to increase the risk of collision, and is motivated by impatience, annoyance, hostility, and/or an attempt to save time [9]; or any driving behavior that intentionally (whether fueled by anger or frustration or as a calculated means to an end) endangers others psychologically, physically, or both [10]. The above definition based on traffic psychology is beneficial for people to understand the causes of ADB, but it is difficult to be directly applied to the recognition of ADB. Therefore, for the accurate recognition of ADB, we define ADB as driving behaviors where a driver intentionally harms another driver in any form, which are typically manifested as abnormal acceleration, abnormal deceleration, abnormal lane change, and tailgating.

In recent years, some studies were conducted on the recognition of ADB, which can be divided into studies based on simulated driving datasets [11,12,13,14] and studies based on naturalistic driving datasets [15,16,17,18,19,20,21,22,23], according to the different datasets utilized. The simulated driving experiment is commonly used in studies on ADB due to its high level of safety. Wang et al. used a semi-supervised support vector machine to divide the driving style between aggressive driving style and normal driving style based on the vehicle dynamic parameters collected in simulated driving experiments [11]. Danaf et al. proposed a hybrid model for aggressive driving analysis and prediction based on the state-trait anger theory, which was verified using simulated driving experiment data [12]. Fitzpatrick et al. studied the influence of time pressure on ADB based on simulated driving experiments [13]. Kerwin et al. concluded that people with high trait anger tend to view many driving behaviors as aggressive, based on the ratings of the videos taken by 198 participants on a driving simulator [14]. Compared with the naturalistic driving experiment, the simulated driving experiment is safer and provides easier control of the experimental conditions. However, there is a certain difference between the data collected through the simulation driving experiment and those collected in the actual traffic environment, which may lead to the problems of low recognition accuracy and high miss rate when the relevant recognition methods are applied to the actual environment. With the development and popularization of vehicle sensor technology and computing platforms, the research on ADB based on naturalistic driving datasets has gradually increased. Ma et al. developed an online approach for aggressive driving recognition using the kinematic parameters that were collected by the in-vehicle recorder under naturalistic driving conditions [15]. Feng et al. verified the performance of the vehicle jerk for recognizing ADB through naturalistic driving data [16].

Both naturalistic driving and simulated driving can provide considerable datasets, which provide the conditions for the studies of ADB recognition based on data-driven methods. Compared with the theory-driven methods, the data-driven methods [17,18,19,20,21,22,23,24,25,26] are naturally suitable for accurately recognizing the complex behaviors in the actual environment because of their capacity to explore the inherent correlations of the captured data warning [27,28] and have been applied in the studies of ADB recognition [17,18,19,20,21,22,23]. Zylius used time and frequency domain features extracted from accelerometer data to build a random forest classifier to recognize aggressive driving styles [17]. Ma et al. used the vehicle motion data collected by the smartphone sensors to compare the recognition performance of the Gaussian mixture model, partial least squares regression, wavelet transformation, and support vector regression on ADB [18]. Carlos et al. used the bag of words method to extract features from accelerometer data and built the models of ADB recognition based on multilayer perceptron, random forest, naive Bayes classifier, and K-nearest neighbor algorithm [19]. Although the recognition of ADB can be realized based on the above methods, a large number of data preprocessing and feature engineering are required in the modeling of time series. In recent years, many deep learning methods have proved to be an effective solution to time series modeling due to their capacity to automatically learn the temporal dependencies present in time series [29]. These deep learning methods have already been applied in the research of ADB recognition [20,21,22,23]. Moukafih et al. proposed a recognition method of ADB based on long short-term memory full convolutional network (LSTM-FCN), and the results showed that the performance of this method is better than some traditional machine learning methods [20]. Matousek et al. realized the recognition of ADB based on long short-term memory (LSTM) and replicator neural network (RNN) [21]. Shahverdy et al. recognized normal, aggressive, distracted, drowsy, and drunk driving styles based on convolutional neural networks (CNN) [22]. Khodairy achieved the recognition of ADB based on stacked long short-term memory (stacked-LSTM) [23].

Although the methods used in the above studies have realized the recognition of ADB, there are still some disadvantages. These methods usually assume that the distribution of the classes in the dataset is relatively balanced and the cost of misclassification is equal. Therefore, these methods cannot properly represent the distribution characteristics of the classes when dealing with class imbalance datasets, which leads to poor recognition performance [30,31,32,33]. Unfortunately, the samples of ADB are usually less than the samples of normal driving behavior (NDB) in the naturalistic driving datasets, which leads these methods to focus on correctly predicting NDB, while ignoring ADB as a minority class. Ensemble learning refers to the methods of training and combining multiple classifiers to complete specific machine learning tasks, which is considered as a solution to the class imbalance problem of machine learning [34]. By combining multiple classifiers, the error of a single classifier may be compensated by other classifiers. Therefore, the recognition performance of the ensemble classifier is usually better than that of a single classifier [34].

According to the above analysis, we propose a recognition method of ADB based on ensemble learning. In this method, the majority class data in the dataset is first divided into multiple groups, and each group of data is combined with the minority class data to construct the class balance dataset; next, the base classifiers are built based on the class balance datasets; finally, the base classifiers are combined based on different ensemble rules to build ensemble classifiers. The salient contributions of our work to the research of ADB recognition can be summarized as follows:

- The acquisition of multi-source naturalistic driving data: combined with the development status of intelligent and connected technology, an integrated experimental vehicle for driving behavior and safety based on the multi-sensor array is constructed. Based on this integrated experimental vehicle, a real vehicle experiment is designed and completed, and a naturalistic driving dataset containing ADB data is acquired;
- The research of the recognition performance of ensemble classifiers: to solve the problem of the poor recognition performance of machine learning method for the ADB data as a minority class in the dataset, a recognition method of ADB based on ensemble learning is proposed.

The rest of this paper is organized as follows. Section 2 introduces the composition of the integrated experimental vehicle for driving behavior and safety, the scheme of the real vehicle experiment, and the method of the data processing. Section 3 introduces the recognition method of ADB based on ensemble learning. Section 4 introduces the comparison results and discussion of the performance of the established ADB identification method and three typical deep learning methods. Section 5 presents the conclusion of this research.

## 2. Data Acquisition and Processing

**Figure 1:**
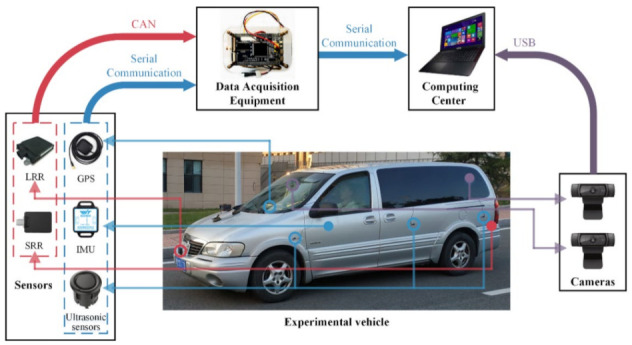
Integrated experimental vehicle for driving behavior and safety.

**Figure 2:**
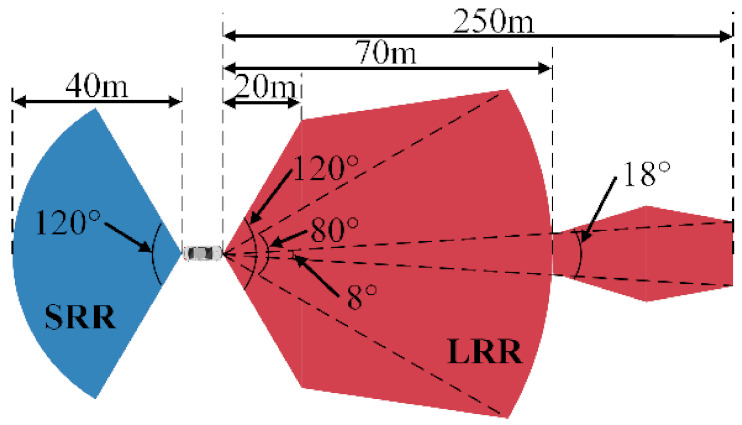
The detection range of the radars.

**Figure 3:**
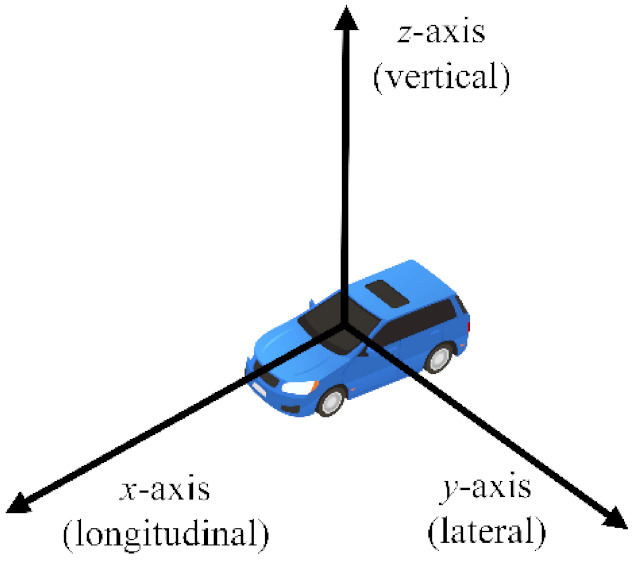
The coordinates of the IMU.

In order to acquire the dataset suitable for the training and verification of the ADB recognition method based on ensemble learning, an integrated experimental vehicle for driving behavior and safety was constructed. As shown in Figure 1, the integrated experimental vehicle consists of the sensors, the data acquisition device, the cameras, the computing center, and the experimental vehicle. The functions of each component of the integrated experimental vehicle are shown in Table 1. The sensors used in the integrated experimental vehicle include long range radar (LRR), short range radar (SRR), inertial measurement unit (IMU), global positioning system (GPS), and an ultrasonic sensor. The functions and installation positions of the above sensors are shown in Table 2. The detection range of the LRR and SRR are shown in Figure 2. The coordinates of the IMU are shown in Figure 3.

The vehicle motion parameters and the driving environment parameters are collected at 10 Hz through the integrated experimental vehicle. The vehicle motion parameters include speed, acceleration, yaw rate, etc. Driving environment parameters include the distance and the relative speed between the integrated experimental vehicle and the objects, etc.

**Figure 4:**
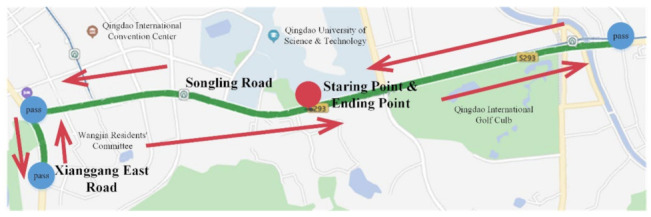
The experimental route of actual driving.

Six consecutive weeks of real vehicle experiment was conducted based on the integrated experimental vehicle for driving behavior and safety. The real vehicle experiment was conducted on one working day and one non-working day every week, and the data acquired every day included the data in rush hour and non-rush hour. Sixteen drivers were selected, including thirteen males and three females, to take part in the experiment. The age distribution of the drivers was between 23 and 50 years, and the average age was 28.9 years old. The driving age distribution was between 2 and 20 years, and the average driving age was 5.3 years. As shown in Figure 4, the road sections of Songling Road-Xianggang East Road in Laoshan, Qingdao were selected as the real vehicle experimental route. The route is a two-direction six-lane urban road with a total length of about 12 km.

According to the aforementioned definition of ADB and previous studies, the longitudinal acceleration, lateral acceleration, yaw rate, distance between the experimental vehicle and the front vehicle, and relative speed between the experimental vehicle and the front vehicle were selected as the features of ADB. The above features are listed in Table 3. Among them, $a_{x}$ is related to abnormal acceleration and deceleration, because the abnormal acceleration and deceleration are usually manifested as large longitudinal acceleration and deceleration; $a_{y}$ and $\omega_{z}$ are related to abnormal lane changes because abnormal lane changes are usually manifested as large lateral accelerations and the large yaw rate; and $d_{f}$ and $v_{f}$ are related to the tailgating.

The essence of ADB recognition is a problem of the time series classification. Therefore, before building the model, a sliding window of fixed length is utilized to segment the data into overlapping series [23,35,36]. The length of the sliding window should be longer than the duration of the four abnormal driving events recorded in our experiment. However, the difference between the ADB series and the NDB series may be reduced if the sliding window is too long, which will lead to an increase in the miss rate and a decrease in the recognition accuracy. To balance the recognition and real-time performance of the recognition method of ADB, a sliding window with 50 time steps and 80% overlap is selected to process the raw data after several iterations of experiments.

Because the features we selected have different scales, the z-score is used to standardize the features, and the definition is shown in Equation (1).

(1) $$z=\frac{x-\mu }{\sigma }$$

where x is the unstandardized data, μ is the mean of the feature vector, σ is the standard deviation of the feature vector, and z is the standardized data.

After the above processing steps, a class imbalance dataset consisting of 31,506 standardized driving behavior series was obtained, which contained 28,908 NDB series and 2598 ADB series.

## 3. Recognition Method

Deep learning methods such as CNN [37], LSTM [38], and gated recurrent unit (GRU) [39] are widely used in time series modeling [40,41,42,43,44] due to their capacity to automatically learn the temporal dependencies present in time series [29]. The essence of ADB recognition is the classification of the time series, so CNN, LSTM, and GRU are utilized in this research. However, the recognition performance of the above methods is also sensitive to the imbalance of classes. Aiming to deal with this problem, an ensemble learning method [45] is employed to realize the recognition of ADB. The ensemble learning method models the class imbalance datasets by transforming one class imbalance problem into multiple class balance problems. The processes are as follows:

(1) Dataset balancing: the majority class data in the dataset are divided into several groups so that the amount of data in each group is similar to that of the minority class data, and then each group of data is combined with the minority data to form multiple class balance datasets.
(2) Base classifiers building: a base classifier is built for each class balance dataset based on a specific classification method.
(3) Ensemble classifiers building: the obtained multiple base classifiers are combined into an ensemble classifier based on ensemble rules.

**Figure 5:**
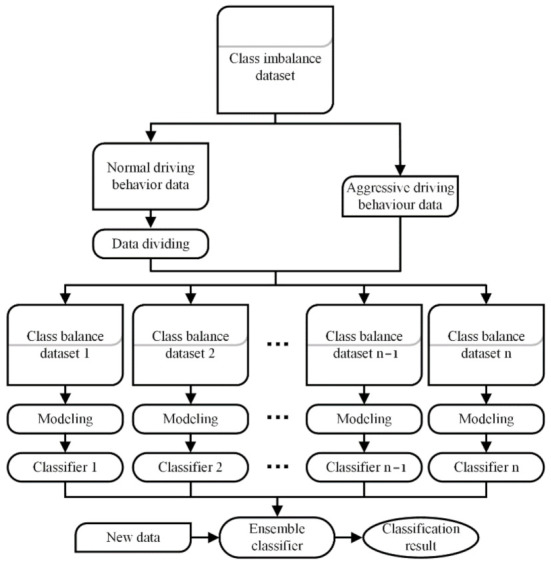
The framework of the recognition method of ADB based on ensemble learning.

The framework of the recognition method of ADB based on ensemble learning is shown in Figure 5.

### 3.1. Dataset Balancing

**Figure 6:**
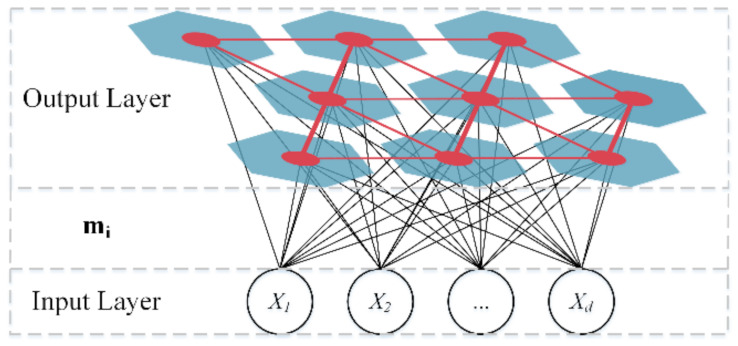
The basic structure of SOM.

In the dataset balancing, the majority class data is divided into multiple groups, and each group of data is combined with the minority data to form multiple class balance datasets. Therefore, a clustering-based dataset balancing method is utilized [45], aiming to make the divided groups of majority class data have a similar amount to the minority class data in the dataset, and make the difference of the data within each group smaller. In this research, the self-organizing map (SOM) [46] is used to cluster the majority class data. SOM is an unsupervised learning neural network method that can map the high-dimension data to low-dimension space and is widely used in various fields, such as emotional intelligence [47], big data analysis [48], water quality assessment [49], and fault prediction [50]. The basic structure of SOM is shown in Figure 6. The output layer of SOM consists of a two-dimensional regular grid of neurons, and each neuron is represented by a weight vector $m_{k}$, $m_{k}=[m_{k1},m_{k2},\ldots ,m_{kd}]$, where d is the dimension of the input data, and kis the number of SOM neurons. In the clustering, SOM assigns the input data to the nearest neuron and updates the weight vector to minimize the distance of the data in the same neuron.

The processes of dataset balancing based on SOM are as follows:

(1) Randomly initialize the weight vectors $m_{k}$.
(2) Input a 5-dimensional NDB series with 50-time steps as a 250-dimensional sample X and calculate the distance between the sample X and the weight vectors $m_{k}$. Calculate the Best-matching unit (BMU) $m_{c}$ according to Equation (2), where BMU is the weight vector closest to the sample X.
  (2) $$\left\|X-m_{c}\right\|=\underset{k}{\min}\left\{\left\|X-m_{k}\right\|\right\}$$
(3) BMU and its topological neighbors are updated according to Equation (3).
  (3) $$m_{k}(T+1)=m_{k}(T)+G_{c,k}(T)(X-m_{k}(T))$$
  (4) $$G_{c,k}(T)=\alpha \left(T\right)\exp\left(-\frac{L_{c,k}^{2}}{2\sigma^{2}\left(T\right)}\right)$$
  where T is the regression steps, $G_{c,k}(T)$ is the neighborhood function, $L_{c,k}$ is the distance between the sample X and BMU $m_{c}$, $\sigma \left(T\right)$ is the neighborhood kernel radius, and $\alpha \left(T\right)$ is the learning rate factor. Both $\sigma \left(T\right)$ and $\alpha \left(T\right)$ decrease monotonically with the regression steps.
(4) Repeat steps (2) and (3) until the training is completed and the samples are divided into k groups.
(5) Combine k groups of NDB samples with ADB samples to form k groups of class balance datasets.

### 3.2. Base Classifiers Building

**Figure 7:**
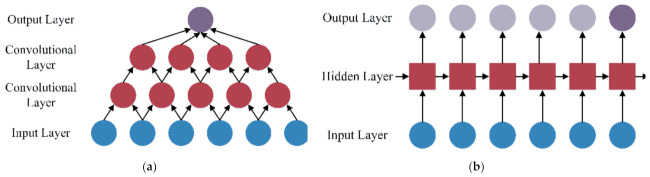
(**a**) The basic structure of CNN; (**b**) the basic structure of RNN.

**Figure 8:**
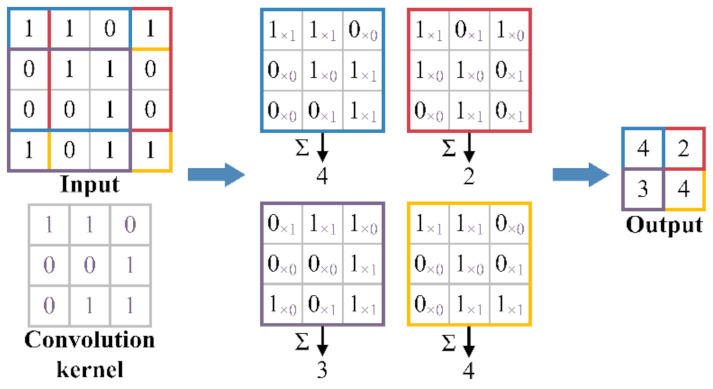
The process of the convolution operation.

**Figure 9:**
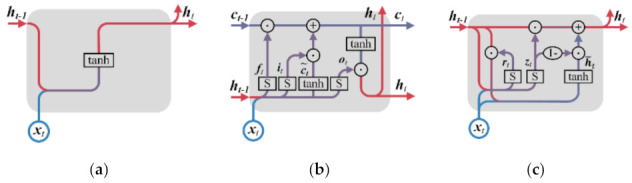
(**a**) The structure of RNN; (**b**) the structure of LSRM; (**c**) the structure of GRU.

After dataset balancing, the deep learning methods of CNN, LSTM, and GRU are employed to build multiple base classifiers with the multiple groups of class balance datasets. CNN is a deep neural network, usually composed of the convolutional layer, pooling layer, and fully connected layer. The basic structure of CNN is shown in Figure 7a. CNN can automatically extract features from high-dimensional raw data with network topology through convolution operations and is often used in machine vision and image processing [51,52]. The convolution operation is a sliding filter, which can capture repetitive patterns in time series through learning. The process of convolution operation is shown in Figure 8. Due to the above characteristics, CNN has been applied in time series modeling, such as financial market prediction [40], natural language processing [41], and driving behavior prediction [22]. CCN performs better than LSTM in some time series modeling tasks [53,54] and has a faster calculation speed [29,53]. LSTM and GRU are two improved recurrent neural networks (RNN), which can solve the problems of gradient disappearance and gradient explosion in traditional RNN when learning long-term dependence. RNN is widely used in time series modeling because it can connect each time step with the previous time step to model the temporal dependencies of time series, such as traffic flow prediction [42], natural language processing [43], and financial market prediction [44]. The basic structure of RNN is shown in Figure 7b. As shown in Figure 9, the difference between RNN, LSTM, and GRU is the hidden layer.

LSTM is widely used in time series modeling tasks in various fields [42,43,44,55]. As shown in Figure 9b, LSTM solves the problem of exploding and vanishing gradients through the cell state $c_{t}$, which stores the long-term memory and retains or deletes the information passing through the hidden layer by the forget gate $f_{t}$, the input gate $i_{t}$, and the output gate $o_{t}$. The definitions of forget gate, input gate, and output gate are as follows:

(5) $$f_{t}=S(w_{f1}h_{t-1}+w_{f2}x_{t}+b_{f})$$

(6) $$i_{t}=S(w_{i1}h_{t-1}+w_{i2}x_{t}+b_{i})$$

(7) $$o_{t}=S(w_{o1}h_{t-1}+w_{o2}x_{t}+b_{o})$$

where S is the sigmoid activation function, and $h_{t-1}$ is the hidden state of time t−1.

The current cell state and hidden state are defined as follows:

(8) $$h_{t}=o_{t}⊙\tanh(c_{t})$$

(9) $$c_{t}=f_{t}⊙c_{t-1}\;+\;i_{t}⊙\tanh({\tilde{c}}_{t})$$

(10) $${\tilde{c}}_{t}=\tanh(w_{c1}h_{t-1}+w_{c2}x_{t}+b_{c})$$

where ⊙ is the element-wise product, and tanh is the tanh activation function.

GRU and LSTM have similar performance, but the GRU is simpler to calculate and implement [39] and has better convergence and generalization [56]. As shown in Figure 9c, the hidden unit of the GRU retains or deletes the input information at the current time $x_{t}$ and the hidden state at the previous time $H_{t-1}$ through the reset gate $r_{t}$ and the update gate $z_{t}$ to achieve the capture of short-term and long-term dependence. The reset gate and update gate are defined as follows:

(11) $$r_{t}=S(w_{r1}H_{t-1}+w_{r2}x_{t}+b_{r})$$

(12) $$z_{t}=S(w_{z1}H_{t-1}+w_{z2}x_{t}+b_{z})$$

The hidden state $H_{t}$ is defined as follows:

(13) $$H_{t}=z_{t}⊙H_{t-1}+(1-z_{t})⊙{\tilde{H}}_{t}$$

(14) $${\tilde{H}}_{t}=\tanh(r_{t}w_{H1}H_{t-1}+w_{H2}x_{t}+b_{H})$$

### 3.3. Ensemble Classifiers Building

After the base classifiers building, the base classifiers are combined into the ensemble classifiers based on different ensemble rules. Referring to the ensemble process of the base classifiers of the ensemble learning method employed in this research [45], 10 different ensemble rules are applied to combine the base classifiers. There are five ensemble rules based on classification probabilities, including Max Rule, Min Rule, Product Rule, Majority Vote Rule, and Sum Rule [57]; and five ensemble rules based on classification probability combined with distance weighting mechanism, including MaxDistance Rule, MinDistance Rule, ProDistance Rule, MajDistance Rule, and SumDistance Rule [45]. The 10 ensemble rules and their strategies are shown in Table 4. The $C_{1}$ and $C_{2}$ are the class labels of data. The $R_{1}$ and $R_{2}$ represent the ensemble rules of the classes $C_{1}$ and $C_{2}$. The $P_{j1}$ represents the probability that the jth classifier classifies the data into $C_{1}$. The $P_{j2}$ represents the probability that the jth classifier classifies the data into $C_{2}$. The $D_{j1}$ represents the average distance between the new data and the data with the class label $C_{1}$ in the jth class balance dataset. The $D_{j2}$ represents the average distance between the new data and the data with the class label $C_{2}$ in the jth class balance dataset.

The definition of the function f(x,y) is shown in Equation (15).

(15) $$f(x,y)=\left\{\begin{array}{c} \begin{array}{cc} 1 & x\geq y \end{array}\\ \begin{array}{cc} 0 & x<y \end{array} \end{array}\right.$$

The final classification result of the data is obtained based on the ensemble rules in Table 4. The data class is $C_{1}$ if $R_{1}\geq R_{2}$, otherwise $C_{2}$.

## 4. Results and Discussion

The validation set is composed of 300 NDB samples and 300 ADB samples, which are randomly selected from NDB samples and ADB samples, respectively. The training set is composed of the remaining 28,608 NDB samples and 2298 ADB samples. After the training is completed, the accuracy (a), precision (p), recall (r), and F_1_-score (F) of each classifier are calculated. The F_1_-score is the harmonic mean of precision and recall, which is closer to the smaller of the two; a high F_1_-score can ensure that both the precision and recall are high [30]. Therefore, the performance of the classifiers in recognizing ADB is evaluated by F_1_-score as the main evaluation metric and accuracy, precision, and recall as the supplementary evaluation metrics. The definition of F_1_-score is shown in Equation (16):

(16) $$F=\frac{2pr}{p+r}$$

The definitions of accuracy, precision, and recall are as follows:

(17) $$a=\frac{TP+TN}{TP+FP+TN+FN}$$

(18) $$p=\frac{TP}{TP+FP}$$

(19) $$r=\frac{TP}{TP+FN}$$

where TP is true positive, TN is true negative, FP is false positive, and FN is false negative.

**Figure 10:**
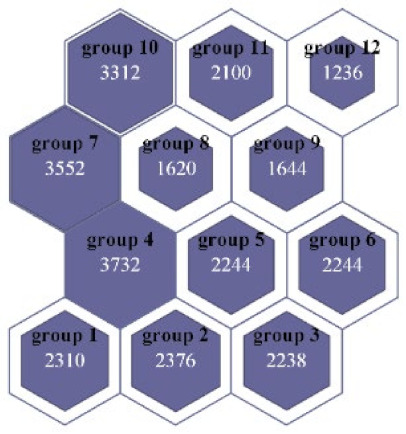
The number of NDB samples in each group.

In the dataset balancing, the NDB samples in the training set are divided into multiple groups, and the number of NDB samples in each group should be as close as possible to the number of ADB samples in the training set. Therefore, according to the ratio of the ADB samples to the NDB samples in the training set, the number of SOM neurons is set as 12, and the mapping size is set to 4 × 3 after several tests. After the clustering, the NDB data in the training set are divided into 12 groups. As shown in Figure 10, the white numbers in grids represent the number of NDB samples in the group. The size of the purple shape in the grid is proportional to the number of NDB samples in this group. By combining the 12 groups of the NDB samples with the ADB samples in the training set, 12 groups of class relative balance datasets are obtained.

**Figure 11:**
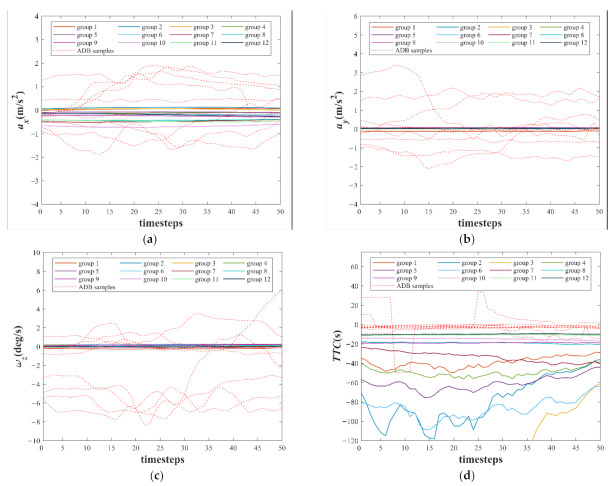
(**a**) The *a_x_* of the weight vectors and ADB samples; (**b**) the *a_y_* of the weight vectors and ADB samples; (**c**) the *ω_z_* of the weight vectors and ADB samples; (**d**) the *TTC* of the weight vectors and ADB samples.

The weight vectors of 12 neurons after training are shown in Figure 11. To show the characteristics of the 12 weight vectors more intuitively, they are compared with several randomly selected ADB samples. As shown in Figure 11a–c, the values and the fluctuations with time steps of the $a_{x}$, the $a_{y}$, and the $\omega_{z}$ of the 12 weight vectors are small, whereas the values and the fluctuations with time steps of the $a_{x}$, the $a_{y}$, and the $\omega_{z}$ of most ADB samples are large. Because the difference between $d_{f}$ and $v_{f}$ of the 12 weight vectors and the ADB samples is difficult to be directly observed, the time to collision (TTC) is calculated based on $d_{f}$ and $v_{f}$. The definition of TTC is shown in Equation (20), and the negative value of TTC means that the experimental vehicle is approaching the vehicle in front. As shown in Figure 11d, the TTC of the 12 weight vectors and some ADB samples is less than 0. However, the TTC of these ADB samples is closer to 0, which means a higher risk of collision.

(20) $$TTC=\frac{d_{f}}{v_{f}}$$

After the dataset balancing, all the methods, including CNN, LSTM, and GRU, are used to build 12 base classifiers with the 12 groups of class balance datasets, respectively, and the 12 base classifiers are combined into the ensemble classifiers based on the 10 different ensemble rules shown in Table 4. In addition, CNN, LSTM, and GRU are used to directly build the classifiers on the class imbalance dataset without ensemble learning. The main parameters of CNN, LSTM, and GRU are shown in Table 5, which are selected after several tests. The number of layers in Table 5 indicates the number of convolutional layers, LSTM layers, or GRU layers in the models. The CNN is designed with a single convolutional layer. LSTM is designed with a single LSTM layer with 128 hidden units. GRU is designed with a single GRU layer with 128 hidden units. In addition, the “/” in Table 5 indicates that the parameter is not utilized in the models.

**Figure 12:**
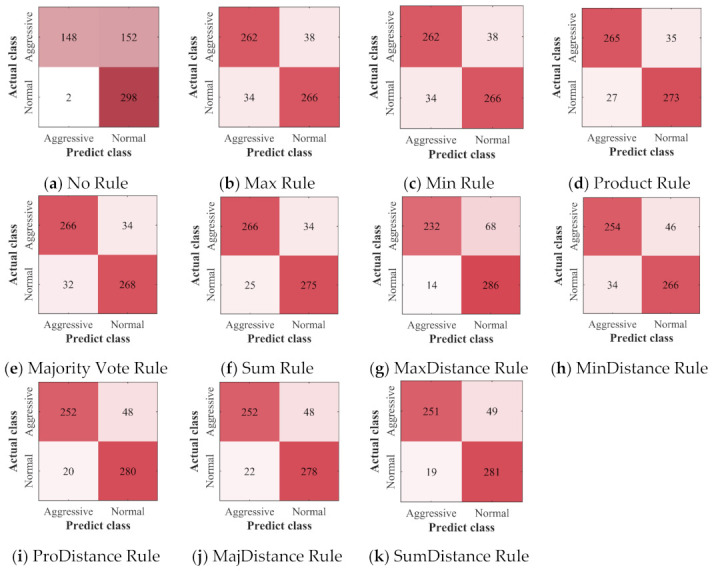
The confusion matrices of CNN: (**a**) the confusion matrix of the classifier built without ensemble learning; (**b**–**k**) the confusion matrices of ensemble classifiers built with 10 different ensemble rules.

**Figure 13:**
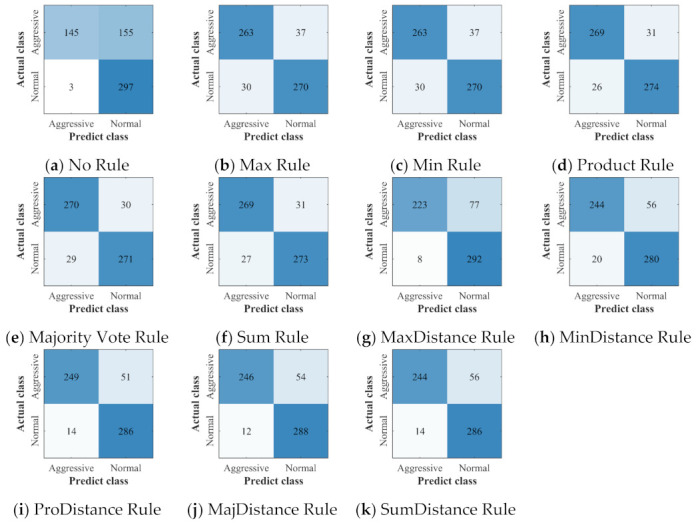
The confusion matrices of LSTM: (**a**) the confusion matrix of the classifier built without ensemble learning; (**b**–**k**) the confusion matrices of ensemble classifiers built with 10 different ensemble rules.

**Figure 14:**
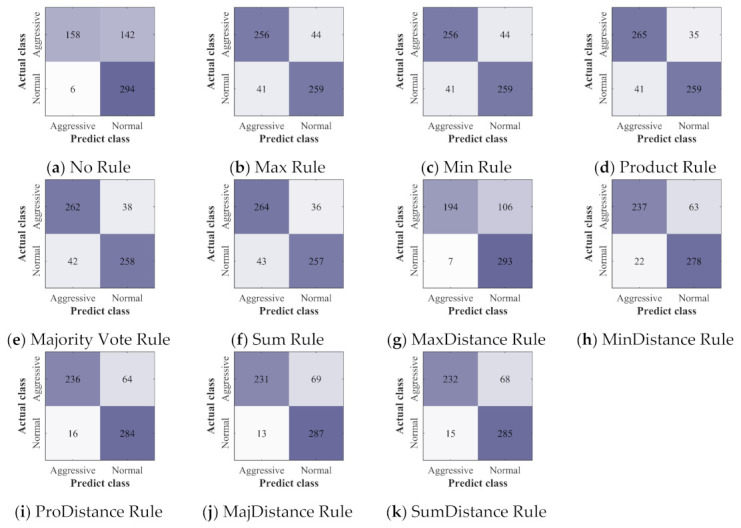
The confusion matrices of GRU: (**a**) the confusion matrix of the classifier built without ensemble learning; (**b**–**k**) the confusion matrices of ensemble classifiers built with 10 different ensemble rules.

The confusion matrices of all the classifiers and ensemble classifiers obtained by verification are shown in Figure 12, Figure 13 and Figure 14. The results obtained by the three deep learning methods before and after the application of ensemble learning have similar characteristics. Compared with the classifier built without ensemble learning, the ensemble classifier built with ensemble learning has a slight increase in the misclassification of NDB samples, but it greatly improves the accuracy of the classification of ADB samples. For the ensemble classifiers, the ones built with the ensemble rules based on classification probability have obtained similar results, and they have fewer misclassification of ADB samples. However, after the ensemble rules based on classification probability are combined with the distance weighting mechanism, they have more misclassification of ADB samples and fewer misclassification of NDB samples.

In order to express the performance of each classifier more intuitively, the accuracy, precision, recall, and F_1_-score of each classifier are calculated and listed in Table 6, Table 7 and Table 8.

As shown in Table 6, Table 7 and Table 8, the ensemble classifiers achieve higher accuracy, recall, and F_1_-score, which shows that compared with classifiers built without ensemble learning, the ensemble classifiers can recognize ADB more accurately. Classifiers built without ensemble learning achieve higher precision and lower recall, which reflects the problem that some machine learning methods are more likely to misclassify minority classes. Compared with the ensemble rules based on classification probability, the ensemble rules combined with the distance weighting mechanism achieve higher precision and lower recall, which means that they have more misclassification of ADB samples. This may be caused by the high dimension of time series data because the distance difference between different data points gradually decreases with the increase of dimension [58]. Therefore, the ensemble classifiers built without distance weighting mechanism are more suitable for the recognition of ADB.

Among the classifiers built without ensemble learning, the one based on the GRU achieves the highest accuracy of 75.33%, recall rate of 52.67%, and F_1_-score of 68.10%, whereas the one based on the CNN achieves the highest precision of 98.66%. Therefore, among the three classifiers built without ensemble learning, the one based on the GRU with the highest F_1_-score achieves the best performance in the recognition of ADB.

Among the ensemble classifiers, the one based on the LSTM and the Product Rule achieves the highest accuracy of 90.50%, which indicates that only 9.50% of the samples are misclassified. The one based on the LSTM and the Majority Vote Rule achieves the highest recall of 90.00%, which indicates that only 10.00% of ADB samples are misclassified. The one based on the LSTM and the MaxDistance Rule achieves the highest precision of 96.54%, which indicates that only 3.46% of the samples classified as ADB are misclassified. The one based on the LSTM and the Product Rule achieves the highest F_1_-score of 90.42%.

Ensemble learning has the greatest improvement to the LSTM, which makes the LSTM ensemble classifiers perform the best in the recognition of ADB. The performance of the CNN ensemble classifiers in the recognition of ADB is second only to the LSTM ensemble classifiers, and the GRU ensemble classifiers have the worst performance.

**Figure 15:**
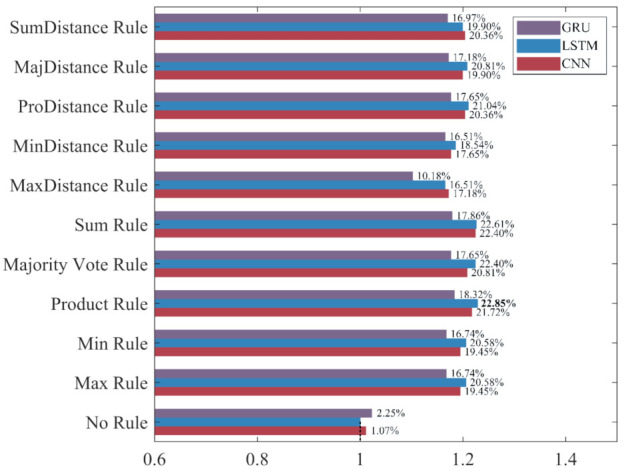
The increase rate and decrease rate of the accuracy for ensemble classifiers. The bold data indicates the highest increase rate.

**Figure 16:**
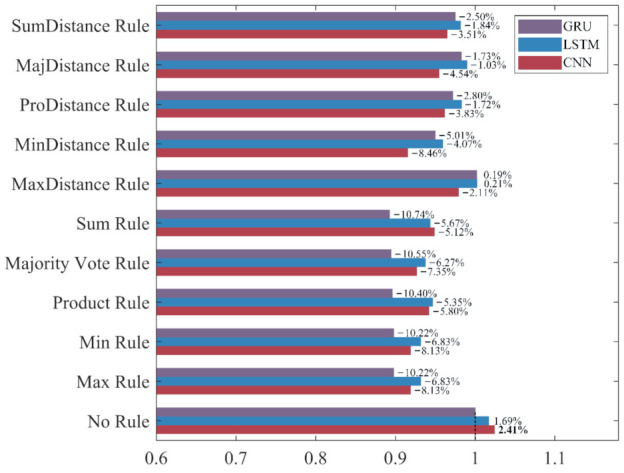
The increase rate and decrease rate of the precision for ensemble classifiers. The bold data indicates the highest increase rate.

**Figure 17:**
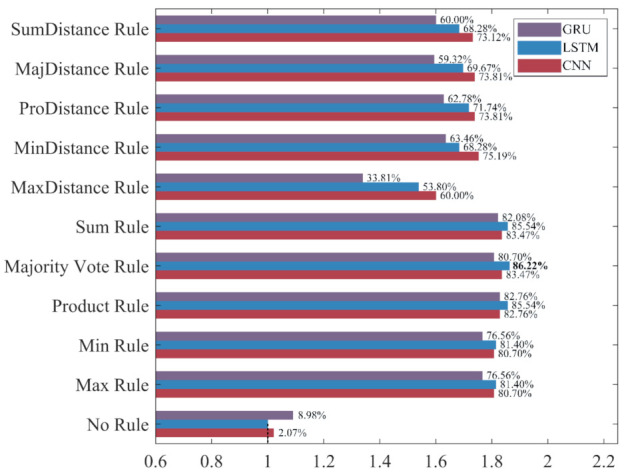
The increase rate and decrease rate of the recall for ensemble classifiers. The bold data indicates the highest increase rate.

**Figure 18:**
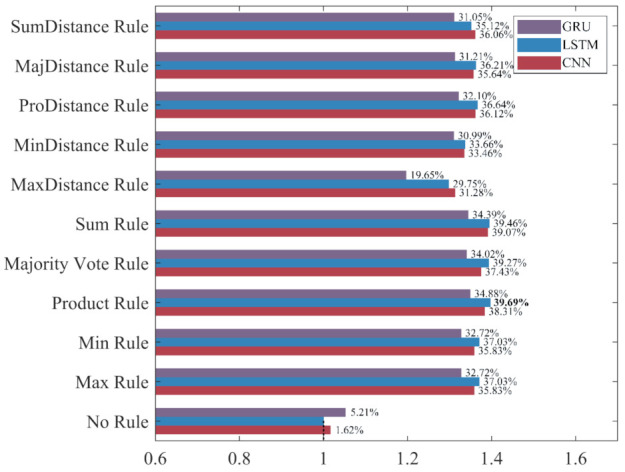
The increase rate and decrease rate of the F_1_-score for ensemble classifiers. The bold data indicates the highest increase rate.

To intuitively compare the influence of different ensemble rules on the recognition performance under different evaluation metrics, the classifier built without ensemble learning, which has the worst performance in each evaluation metric, is used as the benchmark “1” to calculate the increase rate and decrease rate of the evaluation metrics for ensemble classifiers. The results are shown in Figure 15, Figure 16, Figure 17 and Figure 18.

As shown in Figure 15, Figure 16, Figure 17 and Figure 18, the accuracy, recall, and F_1_-score of the three deep learning methods are significantly improved by ensemble learning. As shown in Figure 15, the increase rate of the accuracy for each ensemble classifier is more than 10%, among which the one based on the LSTM and the Product Rule achieves the highest increase rate of 22.85%, followed by the one based on the LSTM and the Sum Rule. This means that although the ensemble learning method increases the misclassification of NDB samples, it reduces the misclassification of more ADB samples. As shown in Figure 16, the precision of most ensemble classifiers has slightly decreased, among which the one based on the GRU and the Sum Rule achieves the highest decrease rate of 10.74%. Although only the ensemble classifiers based on the MaxDistance rule have increased precision, their increase rates of other evaluation metrics are the lowest. Therefore, we consider that the ensemble classifiers based on the MaxDistance Rule have the worst performance. As shown in Figure 17, the increase rate of the recall for each ensemble classifier is more than 33%, among which the one based on the LSTM and the Majority Vote Rule achieves the highest increase rate of 86.22%, followed by the one based on the LSTM and the Product Rule and the Sum Rule. The recall has been significantly improved, which shows that the misclassification of minority class samples is substantially reduced after the application of the ensemble learning method. As shown in Figure 18, the increase rate of the F_1_-score for each ensemble classifier is more than 19%, among which the one based on the LSTM and the Product Rule achieves the highest increase rate of 39.69%, followed by the one based on the LSTM and the Sum Rule. The F_1_-score of most ensemble classifiers is about 30%, which shows that the ensemble learning method effectively improves the recognition performance of three deep learning methods for ADB.

Among the 10 ensemble rules, the Product Rule has the highest improvement to the LSTM and the GRU. Compared with the LSTM and the GRU classifiers built without ensemble learning, the increase rate of the F_1_-score for the LSTM and the GRU ensemble classifier based on the Product Rule are 39.69% and 34.88%, and the LSTM ensemble classifier based on the Product Rule achieves the highest F_1_-score of 90.42%. The Sum Rule achieves the highest improvement to the CNN. Compared with the CNN classifier built without ensemble learning, the increase rate of the F_1_-score for the CNN ensemble classifier based on the Sum Rule is 39.07%. Compared with the other ensemble classifiers, CNN, LSTM, and GRU ensemble classifiers based on the MaxDistance Rule achieves higher precision and lower accuracy, recall, and F_1_-score, and have the worse performance in the recognition of ADB. Overall, ensemble learning significantly improves the recognition performances of the three deep learning methods for ADB. The LSTM ensemble classifier based on the Product Rule with the highest F_1_-score of 90.42% achieves the best performance for ADB recognition, followed by the LSTM ensemble classifier based on the Sum Rule. However, most of the ensemble classifiers built based on ensemble learning have a slight decrease in precision, which means that their recognition performance for NDB has decreased.

In the research, the recognition of ADB is realized based on the motion parameters of vehicles. However, ADB is not only reflected in the four abnormal driving behaviors specified in this research, it is also reflected in behaviors such as frequent whistles and disregard of traffic rules. Therefore, the research on the recognition of aggressive driving behavior that integrates existing parameters and other behavior-related parameters is the focus topic of further work. In addition, verifying the application of other methods in this ensemble learning framework is also the focus of future work, such as applying other clustering methods or directly dividing the majority class samples in the dataset balancing and the application of other deep learning methods in the base classifiers building. Moreover, we will also focus on the application of unsupervised learning and semi-supervised learning methods in the research of aggressive driving behavior recognition.

## 5. Conclusions

The accurate recognition of ADB is the premise to timely and effectively conduct warning or intervention to the driver, which is of great importance for improving driving safety. In this paper, a recognition method of ADB is built based on ensemble learning through the dataset balancing, base classifiers building, and ensemble classifiers building, and the method is trained and verified by a multi-source driving behavior dataset acquired under naturalistic driving conditions. The results suggest that the ensemble classifiers built with ensemble learning achieve higher accuracy, recall, and F_1_-score. In contrast, although the classifiers built without ensemble learning achieve higher precision, they have lower accuracy, recall, and F_1_-score. This comparison result suggests that the ensemble classifier is more suitable for accurately recognizing the ADB with a small proportion in the dataset, whereas the classifier built without ensemble learning is more suitable for recognizing the NDB that is more common. Among the ensemble classifiers built with different rules, the one based on the LSTM and the Product Rule obtains the highest accuracy (90.50%) and F_1_-score (90.42%), which has the optimal performance for ADB recognition. The one based on the LSTM and the Sum Rule has the suboptimal performance for ADB recognition. In summary, the recognition method of ADB based on ensemble learning proposed in this paper can solve the problem of class imbalance in the dataset and achieve a significant improvement in recognition performance. The results can provide a reference for the improvement of the advanced driver assistance system and the realization of personalized driver assistance systems as well as the anthropomorphic automatic vehicle.

## Figures and Tables

**Table 1:** The composition and function of the integrated experimental vehicle.

Compositions	Functions
Sensors	Acquire the vehicle motion parameters and the driving environment parameters.
Data acquisition device	Receive the data acquired by sensors and send it to the computing center.
Cameras	Record the video of the driving environment in the front and rear of the integrated experimental vehicle.
Computing center	Receive and save the data acquired by the data acquisition device and the video recorded by the cameras.
Experimental vehicle	The carrier for the sensors, data acquisition device, cameras, and computing center.

**Table 2:** The functions and installation positions of the above sensors.

Sensors	Functions	Installation Positions
Long range radar	Acquire the distance and relative speed between the integrated experimental vehicle and the front objects.	Above the front bumper.
Short range radar	Acquire the distance and the relative speed between the integrated experimental vehicle and the rear objects.	Above the rear bumper.
Inertial measurement unit	Acquire the acceleration and yaw rate of the vehicle.	About 1.8 m away from the front of the vehicle in the cab.
Global positioning system	Acquire the speed of the vehicle.	Above the console.
Ultrasonic sensor	Acquire the distance between the integrated experimental vehicle and the objects on the left and right sides.	On the left and right sides of the car.

**Table 3:** Features.

Features	Descriptions	Units
$a_{x}$	The acceleration in the x-axis direction of the IMU, that is, the longitudinal acceleration of the vehicle.	m/s^2^
$a_{y}$	The acceleration in the y-axis direction of the IMU, that is, the lateral acceleration of the vehicle.	m/s^2^
$\omega_{z}$	The angular velocity in the z-axis direction of the IMU, that is, the yaw rate of the vehicle.	deg/s
$d_{f}$	The distance between the vehicle and the front vehicle.	m
$v_{f}$	The relative speed between the vehicle and the front vehicle.	m/s

**Table 4:** The strategies of the ensemble rules.

Ensemble Rules	Strategies
Max Rule	$R_{1}=\arg\underset{1<j<K}{\max}P_{j1}$ $,\;R_{2}=\arg\underset{1<j<K}{\max}P_{j2}$
Min Rule	$R_{1}=\arg\underset{1<j<K}{\min}P_{j1}$ $,\;R_{2}=\arg\underset{1<j<K}{\min}P_{j2}$
Product Rule	$R_{1}=\prod_{j=1}^{K}P_{j1}$ $,\;R_{2}=\prod_{j=1}^{K}P_{j2}$
Majority Vote Rule	$R_{1}=\sum_{j=1}^{K}f(P_{j1},P_{j2})$ $,\;R_{2}=\sum_{j=1}^{K}f(P_{j2},P_{j1})$
Sum Rule	$R_{1}=\sum_{j=1}^{K}P_{j1}$ $,\;R_{2}=\sum_{j=1}^{K}P_{j2}$
MaxDistance Rule	$R_{1}=\arg\underset{1<j<K}{\max}\frac{P_{j1}}{D_{j1}+1}$ $,\;R_{2}=\arg\underset{1<j<K}{\max}\frac{P_{j2}}{D_{j2}+1}$
MinDistance Rule	$R_{1}=\arg\underset{1<j<K}{\min}\frac{P_{j1}}{D_{j1}+1}$ $,\;R_{2}=\arg\underset{1<j<K}{\min}\frac{P_{j2}}{D_{j2}+1}$
ProDistance Rule	$R_{1}=\prod_{j=1}^{K}\frac{P_{j1}}{D_{j1}+1}$ $,\;R_{2}=\prod_{j=1}^{K}\frac{P_{j2}}{D_{j2}+1}$
MajDistance Rule	$R_{1}=\sum_{j=1}^{K}\frac{f(P_{j1},P_{j2})}{D_{j1}+1}$ $,\;R_{2}=\sum_{j=1}^{K}\frac{f(P_{j2},P_{j1})}{D_{j2}+1}$
SumDistance Rule	$R_{1}=\sum_{j=1}^{K}\frac{P_{j1}}{D_{j1}+1}$ $,\;R_{2}=\sum_{j=1}^{K}\frac{P_{j2}}{D_{j2}+1}$

**Table 5:** The parameters of models.

Models	Batch Size	Learning Rate	Layers	Units	Convolution				Max Pooling		
					Filters	Filters Size	Stride	Padding Size	Size	Stride	Padding Size
CNN	32	0.001	1	/	10	5 × 2	1	0	2 × 2	2	0
LSTM	32	0.001	1	128	/				/		
GRU	32	0.001	1	128	/				/		

**Table 6:** The validation results of CNN classifiers based on different ensemble rules.

Ensemble Rules	Accuracy	Precision	Recall	F_1_-Score
No Rule	74.33%	98.66%	49.33%	65.78%
Max Rule	88.00%	88.51%	87.33%	87.92%
Min Rule	88.00%	88.51%	87.33%	87.92%
Product Rule	89.67%	90.75%	88.33%	89.53%
Majority Vote Rule	89.00%	89.26%	88.67%	88.96%
Sum Rule	90.17%	91.41%	88.67%	90.02%
MaxDistance Rule	86.33%	94.31%	77.33%	84.98%
MinDistance Rule	86.67%	88.19%	84.67%	86.39%
ProDistance Rule	88.67%	92.65%	84.00%	88.11%
MajDistance Rule	88.33%	91.97%	84.00%	87.80%
SumDistance Rule	88.67%	92.96%	83.67%	88.07%

**Table 7:** The validation results of LSTM classifiers based on different ensemble rules.

Ensemble Rules	Accuracy	Precision	Recall	F_1_-Score
No Rule	73.67%	97.97%	48.33%	64.73%
Max Rule	88.83%	89.76%	87.67%	88.70%
Min Rule	88.83%	89.76%	87.67%	88.70%
Product Rule	90.50%	91.19%	89.67%	90.42%
Majority Vote Rule	90.17%	90.30%	90.00%	90.15%
Sum Rule	90.33%	90.88%	89.67%	90.27%
MaxDistance Rule	85.83%	96.54%	74.33%	83.99%
MinDistance Rule	87.33%	92.42%	81.33%	86.52%
ProDistance Rule	89.17%	94.68%	83.00%	88.45%
MajDistance Rule	89.00%	95.35%	82.00%	88.17%
SumDistance Rule	88.33%	94.57%	81.33%	87.46%

**Table 8:** The validation results of GRU classifiers based on different ensemble rules.

Ensemble Rules	Accuracy	Precision	Recall	F_1_-Score
No Rule	75.33%	96.34%	52.67%	68.10%
Max Rule	86.00%	86.49%	85.33%	85.91%
Min Rule	86.00%	86.49%	85.33%	85.91%
Product Rule	87.17%	86.32%	88.33%	87.31%
Majority Vote Rule	86.67%	86.18%	87.33%	86.75%
Sum Rule	86.83%	85.99%	88.00%	86.99%
MaxDistance Rule	81.17%	96.52%	64.67%	77.45%
MinDistance Rule	85.83%	91.51%	79.00%	84.79%
ProDistance Rule	86.67%	93.64%	78.67%	85.51%
MajDistance Rule	86.33%	94.67%	77.00%	84.93%
SumDistance Rule	86.17%	93.93%	77.33%	84.83%

## Data Availability

The data presented in this study are available on request from the corresponding author. The data are not publicly available due to privacy.
